# Multilook SAR Image Segmentation with an Unknown Number of Clusters Using a Gamma Mixture Model and Hierarchical Clustering

**DOI:** 10.3390/s17051114

**Published:** 2017-05-12

**Authors:** Quanhua Zhao, Xiaoli Li, Yu Li

**Affiliations:** Institute for Remote Sensing Science and Application, School of Geomatics, Liaoning Technical University, Fuxin 123000, China; lixiaolilntu@163.com (X.L.); liyu@lntu.edu.cn (Y.L.)

**Keywords:** hierarchical clustering, Gamma mixture model (GaMM), unknown number of clusters, SAR image segmentation, Markov Random Field (MRF)

## Abstract

This paper presents a novel multilook SAR image segmentation algorithm with an unknown number of clusters. Firstly, the marginal probability distribution for a given SAR image is defined by a Gamma mixture model (GaMM), in which the number of components corresponds to the number of homogeneous regions needed to segment and the spatial relationship among neighboring pixels is characterized by a Markov Random Field (MRF) defined by the weighting coefficients of components in GaMM. During the algorithm iteration procedure, the number of clusters is gradually reduced by merging two components until they are equal to one. For each fixed number of clusters, the parameters of GaMM are estimated and the optimal segmentation result corresponding to the number is obtained by maximizing the marginal probability. Finally, the number of clusters with minimum global energy defined as the negative logarithm of marginal probability is indicated as the expected number of clusters with the homogeneous regions needed to be segmented, and the corresponding segmentation result is considered as the final optimal one. The experimental results from the proposed and comparing algorithms for simulated and real multilook SAR images show that the proposed algorithm can find the real number of clusters and obtain more accurate segmentation results simultaneously.

## 1. Introduction

Synthetic Aperture Radar (SAR) is widely used in civil and military fields because of its characteristics such as high resolution, strong penetration, full time and full weather [1]. However, its specific imaging mechanism leads to speckle noises in SAR images. Although the multilook technique can partly reduce the type of noises, there are still obvious noises remaining in SAR images [2]. Image segmentation, as a crucial step for image analysis and interpretation, involves separating pixels into clusters, so that the pixels in the same cluster are homogeneous and the pixels in different clusters are heterogenous [3]. The main tasks for image segmentation are to determine a suitable number of clusters and obtain an accurate segmentation result [4]. Due to the inherent speckle noise existing in SAR images, these tasks are difficult to fulfill. Therefore, the objective of the proposed algorithm is to obtain the real number of clusters and the accurate segmentation result simultaneously for speckled SAR images. In this paper, the real number is regarded as the number selected by human visual judgment.

Many approaches have been studied to determine the number of clusters, such as clustering methods [5,6,7,8,9] and statistic methods [10,11,12,13,14]. The most popular clustering method is Iterative Self-Organizing Data Analysis Technique Algorithm (ISODATA) [5], in which the number of clusters is changed by alternately splitting and merging clusters. However, it requires a lot of parameters given by humans, and the K-means dominating optimization in ISODATA algorithm is not suitable for segmenting SAR images because of the statistical characteristics of the speckle noises. In order to reduce the human influence, another clustering method, the hierarchical clustering included agglomerative procedure (bottom-up) and divisive procedure (top-down) [7], was considered. The agglomerative produce is the most commonly used one and the representative algorithm is Agglomerative Nesting (AGNES) [8], which considers each element as a cluster and the two clusters which are closest to each other with minimum distance are merged at each step. This procedure continues until only one cluster exists. In order to overcome the sensitivity of noises to Euclidean distance, Legendre et al. [9] used the average distance to measure the closeness between two clusters, but the smaller cluster is easily ignored. Generally, statistical methods are considered to be a more appropriate strategy in SAR image segmentation [10,11]. Green [12] proposed the Reversible Jump Markov Chain Monte Carlo (RJMCMC) algorithm which has been successfully applied to image segmentation with an unknown number of clusters. Based on the work of Green, Kato [13] combined a mixture model and RJMCMC to complete the segmentation. It regards the number of clusters as a random variable contained in model parameters. The number jumps by sampling operation such as birth or death class, and the optimal number is obtained by maximizing or minimizing the objective function. However, the statistical methods for image segmentation with unknown number of clusters are too cumbersome because the dimension of parameters always changes along with the changing of the number of clusters, and it easily falls into the local optimum. To sum up, hierarchical clustering is convenient for obtaining the number of clusters, and the statistical model can describe images well [14], so hierarchical clustering combining a statistical model is regarded as a more appropriate approach in SAR image segmentation in this paper.

There are many approaches of hierarchical theory with statistical models. Mignotte et al. [15] proposed an unsupervised segmentation method for sonar images based on a hierarchical Markov Random Field (MRF) model; it defines a causal energy function with a Gibbs distribution form. Although it can avoid the ignoring of clusters with a few pixels, it cannot be applied to SAR image segmentation in the absence of the capability to manage the statistical property of the speckle noises. Upgrading the MRF model, Wang et al. [16] proposed a new unsupervised method based on a hierarchical Triplet Markov Field (TMF) model with Gaussian distribution to segment SAR images. It combines the advantages of both hierarchical and TMF in the discrete wavelet domain to deal with the problem of insufficient local statistical information. However, the unimodal characteristic model is not as flexible as a mixture model [17]. Garcia et al. [18] proposed a hierarchical Gaussian Mixture Model (GMM) algorithm, which is able to automatically learn the optimal number of components for the simplified GMM and successfully segment natural images, but the GMM cannot describe the data with non-Gaussian statistical character [19], such as SAR images. Oliver et al. [20] has proved that the intensities of pixels satisfy a Gamma distribution according to the speckle noise existing in SAR images, Dong et al. [21] fully validated that the Gamma distribution with MRF is more suitable for SAR image segmentation than a Gaussian distribution with MRF, so the hierarchical clustering based on a Gamma mixture model (GaMM) is proposed for SAR image segmentation.

In this paper, an over-segmentation result with the number of clusters which is much larger than the real number is given as the initial status. The number of clusters is reduced gradually by a cluster merging operation until the optimal number of clusters is obtained. Firstly, the original image is divided into many subsets in gray level by a span parameter. Each subset is regarded as a cluster, and the two subsets with minimum global energy are merged until the number of clusters is equal to one. In addition, the segmentation model with current number of clusters is established by marginal probability distribution based on the GaMM. The negative logarithm of marginal probability is described as the global energy function, and the smaller the value is, the better the segmentation is. Furthermore, in order to consider the influence of neighboring pixels, the prior probability is defined by the conditional probability of the membership based on MRF. Then, the segmentation results with the current number of clusters can be obtained by posterior probability according to the Bayesian theory [22], the number of clusters reduces by one after each iteration, and a segmentation result and a global energy are obtained under the current number of clusters. Finally, the optimal number of clusters is the one corresponding to the minimum global energy, and the result corresponding to the optimal number is the final optimal segmentation result.

## 2. Description of the Proposed Algorithm

Given a multilook SAR image ***x*** = {*x_i_*, *i* = 1, ..., *n*}, where *i* is the index of pixels, *n* is the number of pixels, *x_i_* is the intensity of pixel *i.* Assume that the image has *m* clusters, in order to represent the image segmentation, membership matrix ***z*** = [*z_ij_*]*_n×m_* is used, where *j* is the index of clusters, *z_ij_* ∈ {0, 1}, $\sum_{j=1}^{m}z_{ij}=1$, *z_ij_* = 1 indicates the *i*th pixel belongs to *j*th cluster. Furthermore, ***z*** can be re-expressed as ***z*** = (***z****_i_*_·_, *i* = 1, …, *n*)^Τ^ = (***z***_·*j*_, *j* = 1, ..., *m*), where ***z****_i_*_·_ = (*z_ij_*, *j* = 1, ..., *m*) and ***z***_·*j*_ = (*z_ij_*, *i* = 1, ..., *n*)^Τ^, where ***z****_i_*_·_ and ***z***_·*j*_ are row and column vectors of membership matrix ***z***, respectively. In the image segmentation, ***x*** is observable data, ***z*** can be considered as unobservable data, {***x***, ***z***} is the complete data set. The image segmentation is the process of estimating unobservable data ***z*** given ***x***.

### 2.1. Segmentation Process

Assume that the conditional distribution of *i*th pixel belonging to *j*th cluster is Gamma distribution and its conditional probability density function (pdf) is:

(1) $$p(x_{i}|z_{ij}=1;\;\theta_{j})=\mathrm{Ga}(x_{i};\;\alpha_{j},\beta_{j})=\frac{x_{i}^{\alpha_{j}-1}}{\Gamma (\alpha_{j})\beta_{j}^{\alpha_{j}}}\exp\left(-\frac{x_{i}}{\beta_{j}}\right)$$

where Ga(·) is the pdf of Gamma distribution, ***θ****_j_* = (*α_j_*, *β_j_*) is distribution parameter vector of *j*th cluster, *α_j_* and *β_j_* are shape and scale parameters of Gamma distribution, respectively.

According to the Bayesian theory, the joint pdf of *x_i_* and *z_ij_* is:

(2) $$p(x_{i},z_{ij};\;\theta_{j})=p(z_{ij}=1)p(x_{i}|z_{ij}=1;\;\theta_{j})$$

The marginal probability of *x_i_* is:

(3) $$p(x_{i};\;\theta )=\sum_{j=1}^{m}p(z_{ij}=1)p(x_{i}|z_{ij}=1;\;\theta_{j})$$

where ***θ*** = {***θ****_j_*, *j* = 1, …, *m*}.

Assume that the marginal distribution of intensity in the SAR image ***z*** is independent of each other, then the marginal probability of image ***x*** is:

(4) $$p(x;\;\theta )=\prod_{i=1}^{n}p(x_{i};\;\theta )=\prod_{i=1}^{n}\sum_{j=1}^{m}p(z_{ij}=1)p(x_{i}|z_{ij}=1;\;\theta_{j})$$

The prior probability for *z_ij_* = 1 is denoted *p*(*z_ij_* = 1) = *π_ij_*, where *π_ij_* ∈ [0, 1] and satisfies $\sum_{j=1}^{m}\pi_{ij}=1$. In order to consider the spatial interaction from neighborhood pixels, the prior probability is defined by a conditional distribution:

(5) $$\pi_{ij}=p(z_{ij}=1|z_{i\prime j}\in N_{i})=\frac{\exp\left(\eta \sum_{i\prime \in N_{i}}z_{i\prime j}\right)}{\sum_{j\prime =1}^{m}\exp\left(\eta \sum_{i\prime \in N_{i}}z_{i\prime j\prime }\right)}$$

where *N_i_* is the neighborhood pixels set of *i*th pixel, *η* is the coefficient indicates the influence of intensities of neighbor pixels, the higher the coefficient *η* is, the stronger the neighbor influence is.

Combining Equations (4) and (5), the marginal probability can be reformed as:

(6) $$p(x;\;\theta )=\prod_{i=1}^{n}\sum_{j=1}^{m}\pi_{ij}\mathrm{Ga}(x_{i};\;\alpha_{j},\beta_{j})$$

As described in Equation (6), the marginal distribution of observable data ***x*** is GaMM, and the prior probability *π_ij_* is the model weight. The number of clusters is equal to the number of GaMM components.

Equation (6) is regarded as the segmentation model expressed by GaMM. The higher the marginal probability is, the better the segmentation result is. There are many criteria to determine the optimal segmentation. Among them, the maximum posterior probability is one of the most commonly used ones. According to the Bayesian theory, the posterior probability of *i*th pixel belonging to *j*th cluster is:

(7) $$p(z_{ij}=1|x_{i};\;\theta )=\frac{p(z_{ij}=1)p(x_{i}|z_{ij}=1;\;\theta_{j})}{\sum_{j\prime =1}^{m}p(z_{ij\prime }=1)p(x_{i}|z_{ij\prime }=1;\;\theta_{j\prime })}=\frac{\pi_{ij}\mathrm{Ga}(x_{i};\;\alpha_{j},\beta_{j})}{\sum_{j\prime =1}^{m}\pi_{ij\prime }\mathrm{Ga}(x_{i};\;\alpha_{j\prime },\beta_{j\prime })}$$

Finally, the optimal segmentation corresponds to the resolution of the maximum posterior probability and can be expressed as:

(8) $$z_{ij}=\left\{ \begin{array}{l} 1\;\mathrm{if}\;p(z_{ij}=1|x_{i};\;\theta_{j})=\max\left\{p(z_{ij\prime }=1|x_{i};\theta_{j\prime }),\;j\prime =1,\;\ldots ,\;m\right\}\\ 0\;\mathrm{otherwise} \end{array}\right.$$

From Equation (8) one can see that the segmentation is the problem of solving the model parameters, ***θ****_j_* = {*α_j_*, *β_j_*}. For multilook SAR images, the shape parameter *α_j_* is usually equal to the number of looks. For scale parameter *β_j_*, it is difficult to solve by Equation (6), thus, the global energy function *L* defined as the negative logarithm of marginal probability of ***x*** is used instead of Equation (6) to evaluate the quality of results. The smaller the function value is, the bigger the marginal probability is, and the better the segmentation result is:

(9) $$L=-\log\;p(x;\;\theta )=-\log\prod_{i=1}^{n}\sum_{j=1}^{m}\pi_{ij}\mathrm{Ga}(x_{i};\;\beta_{j})=-\sum_{i=1}^{n}\log\left\{\sum_{j=1}^{m}\pi_{ij}\mathrm{Ga}(x_{i};\;\beta_{j})\right\}$$

Because the scale parameter *β_j_* is differentiable function of global energy *L*, the derivative of *L* for *β_j_* is:

(10) $$\begin{array}{ll} \frac{\partial L}{\partial \beta_{j}} & =-\sum_{i=1}^{n}\frac{1}{\sum_{j=1}^{m}\pi_{ij}\mathrm{Ga}(x_{i};\;\beta_{j})}\cdot \pi_{ij}\frac{\partial \mathrm{Ga}(x_{i};\;\beta_{j})}{\partial \beta_{j}}\\ & =-\sum_{i=1}^{n}\frac{\pi_{ij}\mathrm{Ga}(x_{i};\;\beta_{j})}{\sum_{j=1}^{m}\pi_{ij}\mathrm{Ga}(x_{i};\;\beta_{j})}\cdot \left(-\frac{\alpha }{\beta_{j}}+\frac{x_{i}}{{\beta_{j}}^{2}}\right)\\ & =-\frac{1}{\beta_{j}}\sum_{i=1}^{n}p(z_{ij}=1|x_{i},\theta_{j})\cdot \left(-\alpha +\frac{x_{i}}{\beta_{j}}\right) \end{array}$$

Let Equation (10) be equal to 0, its solution is equivalent to the solution of Equation (11):

(11) $$\sum_{i=1}^{n}p(z_{ij}=1|x_{i},\;\beta_{j})\cdot \left(-\alpha +\frac{x_{i}}{\beta_{j}}\right)=0$$

It is difficult to solve Equation (11), because *β_j_* exists in both of the first and second term. In order to obtain the best approximate solution, let *β_j_* in the first term equal to the value of the last iteration expressed as *t*, Equation (11) can be rewritten as:

(12) $$\sum_{i=1}^{n}p({z_{ij}}^{\left(t\right)}=1|x_{i},\beta_{j}^{\left(t\right)})\cdot \left(-\alpha +\frac{x_{i}}{\beta_{j}^{\left(t+1\right)}}\right)=0$$

Then the scale parameter $\beta_{j}^{\left(t+1\right)}$ can be solved as:

(13) $${\beta_{j}}^{\left(t+1\right)}=\frac{\sum_{i=1}^{n}p({z_{ij}}^{\left(t\right)}=1|x_{i};{\beta_{j}}^{\left(t\right)})x_{i}}{\sum_{i=1}^{n}p({z_{ij}}^{\left(t\right)}=1|x_{i};{\beta_{j}}^{\left(t\right)})\alpha }$$

The optimization process of the segmentation results under fixed *m* will be stopped when *t* reaches the maximum value of *T*. According to our experiments, it is found that taking *T* = 20 is suitable for all testing images, so in the proposed algorithm, set *T* = 20 as the iterative stopping condition.

In summary, if the initial segmentation *z_ij_*^(0)^ under the current number of clusters *m* is known, the segmentation process regarded as an inner loop under fixed number of clusters is described as follows:

1. Calculating the prior probability *π_ij_*^(*t*)^ by Equation (5);
2. Calculating the scale parameter *β_j_*^(*t*)^ by Equation (13), and calculating conditional probability by Equation (1);
3. Calculating the posterior probability *p*(*z_ij_* = 1|*x_i_*; ***θ****_j_*)^(*t*)^ by Equation (7);
4. Repeating Step 1–3 until *t* is equal to *T*;
5. The optimal segmentation result *z_ij_*^(*T*)^ under fixed number of clusters *m* is obtained by Equation (8).

### 2.2. Hierarchical Clustering Process

In the above Subsection, the optimal segmentation can be obtained under a fixed number of clusters. In order to obtain the optimal result with a real number of clusters, the number of clusters *m* is viewed as a variable in the proposed hierarchical clustering algorithm. The number *m* gradually decreases from an initial number which is much greater than the real number of clusters to one by a cluster merging operation. Thus, one can get the optimal results under each number of clusters by inner loop. At last, the optimal segmentation is determined from the series of optimal results by minimizing global energy defined by Equation (9).

If *τ* is an iterative indicator in the outer loop for hierarchical clustering process, the key point of the hierarchical clustering process is to obtain the initial parameters at the *τ* + 1 iteration, such as, ***z***^(*τ*+1, 0)^, *p*(*z_ij_* = 1|*x_i_*; ***θ****_j_*)^(*τ*+1, 0)^. Further, the optimal results at *τ* + 1 iteration ***z***^(*τ*+1, *T*)^ can be obtained by inner loop. If ***z***_·*j*_ = {***x****_i_*; *z_ij_* = 1}(all pixels in *j*th cluster) expresses the collection of *j*th class, and the segmentation result in *τ* iterative is ***z***^(*τ*, *T*)^ = {***z***_·1_^(*τ*, *T*)^, ..., ***z***_·*j*−1_^(*τ*, *T*)^, ***z***_·*j*_^(*τ*, *T*)^, ***z***_·*j*+1_^(*τ*, *T*)^, ..., ***z***_·*j*’−1_^(*τ*, *T*)^, ***z***_·*j*’_^(*τ*, *T*)^, ***z***_·*j*’+1_^(*τ*, *T*)^, ..., ***z***_·*m*_^(*τ*, *T*)^}. Assuming that the merged clusters are *j* and *j*’, then, ***z***_(*jj’*)_^(*τ*, *)^ = {***z***_·1_^(*τ*, *)^, ..., ***z***_·*j*−1_^(*τ*, *)^, ***z***_·*j*_^(*τ*, *)^, ***z***_·*j*+1_^(*τ*, *)^, ..., ***z***_·*j*’−1_^(*τ*, *)^, ***z***_·*j*’_^(*τ*, *)^, ***z***_·*j*’+1_^(*τ*, *)^, ..., ***z***_·*m*−1_^(*τ*, *)^} ,where ***z***_·*j*_^(*τ*, *)^ = ***z***_·*j*_^(*τ*, *T*)^ + ***z***_·*j*’_^(*τ*, *T*)^, others stay the same and {***z***_·*j*’_^(*τ*, *)^, ***z***_·*j*’+1_^(*τ*, *)^, ..., ***z***_·*m*−1_^(*τ*, *)^} are the rearrange of {***z***_·*j*’+1_^(*τ*, *T*)^, ..., ***z***_·*m*_^(*τ*, *T*)^}. Further, the new global energy function by merging *j* and *j*’ is expressed as *L_jj_*_’_^(*τ*, *)^, let Δ***L***^(*τ*)^ = *L_jj_*_’_^(*τ*, *)^, *j* = 1, ..., *m* − 1, *j*’ = *j* + 1, ..., *m*, choose the clusters $\hat{j}$ and $\hat{j}\prime$ which corresponding to the minimum global energy function, that is:

(14) $$\left\{\hat{j},\hat{j}\prime \right\}=\arg\min\left\{\Delta L^{\left(\tau \right)}:{L_{jj\prime }}^{\left(\tau ,\;*\right)},j=1,\ldots ,m-1,j\prime =j+1,\ldots ,m\right\}$$

The segmentation result in *τ* + 1 iteration is $z^{\left(\tau +1,\;0\right)}={z_{\left(\hat{j}\hat{j}\prime \right)}}^{\left(\tau ,\;*\right)}$, let *m*^(*τ*+1)^ = *m*^(*τ*)^ − 1, and the initial posterior probability in *τ* + 1 iteration *p*(*z_ij_* = 1|*x_i_*; ***θ****_j_*)^(*τ*+1, 0)^ is:

(15) $$p{\left(z_{ij}=1|x_{i};\;\theta_{j}\right)}^{\left(\tau +1,\;0\right)}=p{\left(z_{i\hat{j}}=1|x_{i};\;\theta_{\hat{j}}\right)}^{\left(\tau ,\;T\right)}+p{\left(z_{i\hat{j}\prime }=1|x_{i};\;\theta_{\hat{j}\prime }\right)}^{\left(\tau ,\;T\right)}$$

where $p{\left(z_{i\hat{j}\prime }=1|x_{i};\;\theta_{\hat{j}\prime }\right)}^{\left(\tau +1,\;0\right)}$ does not exist now.

After merging, ***z***^(*τ*+1, *T*)^ can be obtained by inner loop and the global energy function value *L*^(*τ*+1)^ is recorded by new parameters *π_ij_*^(*τ*+1, *T*)^, *β_j_*^(*τ*+1, *T*)^ at each iteration, then the real number of clusters $\hat{m}$ can be estimated by:

(16) $$\left\{\hat{m},\hat{\tau }\right\}=\arg\max\left\{L^{\left(\tau \right)},\tau =0,\;\ldots ,\;m^{\left(0,\;0\right)}\right\}$$

The final optimal segmentation is:

(17) $${\hat{z}}_{ij}={z_{ij}}^{\left(\hat{\tau },\;T\right)}$$

In summary, an initial segmentation must be given before the process of both segmentation and hierarchical clustering. The initial segmentation ***z***^(*τ*=0, *t*=0)^ is given by dividing the image ***x*** into many sub-classes using span parameter *d*. Then the *i*th pixel in *j*th cluster can be expressed as:

(18) $${z_{ij}}^{\left(0,\;0\right)}=\left\lceil \frac{x_{i}}{d}\right\rceil$$

where ⌈·⌉ expresses for rounding up to an integer. The *j*th cluster will be deleted if there are no pixels in it until all clusters are assigned a number of pixels. Then, *m*^(0, 0)^ is equal to the column number of ***z***^(0, 0)^, and the initial scale parameter *β_j_*^(*τ*=0, *t*=0)^ is calculated by:

(19) $${\beta_{j}}^{\left(0,\;0\right)}=\frac{1}{\alpha n_{j}}\sum_{x_{i}\in {z_{\cdot j}}^{\left(0,0\right)}}x_{i}$$

where *n_j_* is the number of pixels in *j*th cluster.

### 2.3. General Procedure of the Proposed Algorithm

The proposed algorithm uses the outer loop with hierarchical clustering to merge clusters in order to find the optimal number of clusters and the inner loop with the GaMM as the segmentation model to obtain the optimal segmentation under current number of clusters. The outer loop stops until the class number is reduced to one. The optimal segmentation under the estimated real number of clusters can be obtained by minimizing the global energy defined in Equation (9).

The process of the proposed algorithm can be summarized as follows:

1. *Initialization*. The iterative indicator of inner loop *t* = 0 and outer loop τ = 0, the maximum number of inner loop *T* = 20 according to the experimental experience, shape parameter *α* which is equal to the looks of the SAR images, intensity of neighbor influence *η* within the range of 0~1;
2. *Coarse segmentation*. The initial segmentation *z_ij_*^(0, 0)^ is obtained by Equation (18), and the initial scale parameter *β_j_*^(0, 0)^ is calculated by Equation (19).
3. *Inner loop*. Calculating the prior probability *π_ij_*^(*τ*, *t*)^ by Equation (5), calculating the posterior probability *p*(*z_ij_* = 1|*x_i_*; ***θ****_j_*)^(*τ*, *t*)^ by Equation (7). In the inner iteration, the scale parameter *β_j_*^(*τ, t*)^ is calculated by Equation (13), after *T* inner iterations, the segmentation *z_ij_*^(*τ*, *T*)^ under the current outer loop is obtained and *L*^(*τ*)^ is recorded by Equation (9);
4. *Merging clusters*. Choosing clusters *j* and *j*’ by Equation (14) which correspond to the minimum global energy function. Merging clusters *j* and *j*’, and updating the parameters of *π_ij_*^(*τ*, *)^, *β_j_*^(*τ*, *)^ by Equations (5) and (13), respectively;
5. *Updating parameters*. After merging, let *m*^(*τ*+1)^ = *m*^(*τ*)^ − 1, and *β_j_*^(*τ*+1, 0)^ is obtained by Equation (15), then the segmentation *z_ij_*^(*τ*+1, *T*)^ and global energy *L*^(*τ*+1)^ are obtained by inner loop;
6. Repeat Step 4–5, and stop the outer loop when *m*^(*τ*)^ = 1;
7. The real number of clusters $\hat{m}$ and final optimal segmentation result ${\hat{z}}_{ij}$ is found by Equations (16) and (17).

## 3. Experimental Results and Discussion

The proposed algorithm is tested with a simulated SAR image and four real SAR images. In addition, the comparing algorithms, including ISODATA [5], improved AGNES [8], HMRF-FCM [6] and Gamma-MRF [21] algorithms are also used to evaluate the proposed algorithm.

### 3.1 Simulated SAR Image Segmentation

**Figure 1:**
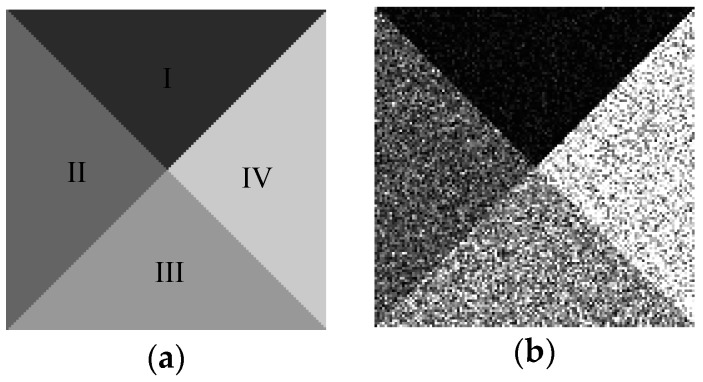
(**a**) Template; (**b**) Simulated image.

Figure 1a is a template with size of 128 × 128 pixels for generating a simulated SAR image which covers four homogeneous regions labeled I-IV, respectively. Figure 1b is the simulated image, in which the intensities of pixels in each homogeneous region are drawn from a Gamma distribution with the shape and scale parameters listed in Table 1.

**Figure 2:**
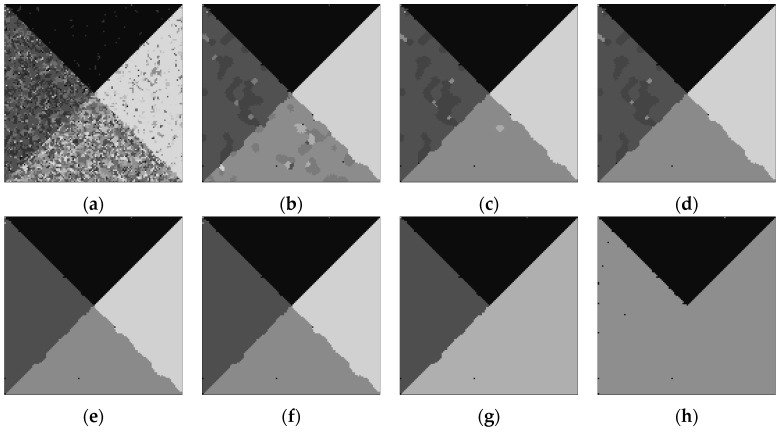
Segmentation process images in iterations. (**a**) Initial, *m* = 9; (**b**) *τ* = 1, *m* = 8; (**c**) *τ* = 2, *m* = 7; (**d**) *τ* = 3, *m* = 6; (**e**) *τ* = 4, *m* = 5; (**f**) *τ* = 5, *m* = 4; (**g**) *τ* = 6, *m* = 3; (**h**) *τ* = 7, *m* = 2.

**Figure 3:**
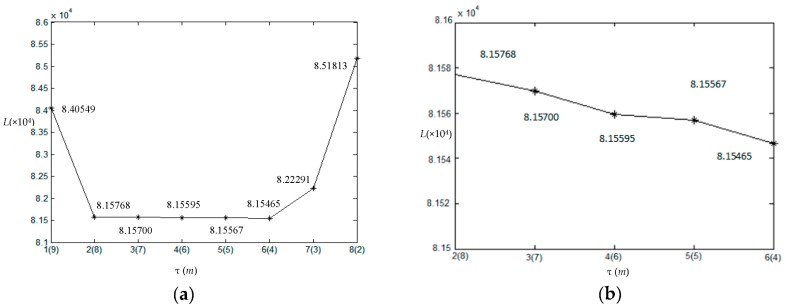
Global energy value *L* and the number of clusters *m* at each iteration. (**a**) For the whole *m*; (**b**) For part of *m* amplification.

In order to generate initial segmentation of image shown in Figure 1b, we set span parameter *d* = 30. Then the initial number of clusters *m* is ⌈255/30⌉ which is equal to 9. In this experiment, *α* = 4 as the looks of SAR image. The coefficient *η*, which expresses the influence of neighbor pixels, is always taken from 0 to 1. If *η* is too small, the results will exist many misclassification pixels although the number of clusters is correct, contrarily, it will be over segmented. According to experiments, *η* = 0.8 which is suitable for the simulated images. Figure 2a is the initial segmentation with *m* = 9. Figure 2b–h show the corresponding segmentation results with the gradually decreased number of clusters during outer loop. It shows that the initial segmentation in gray level already can roughly distinguish each homogeneous regions shown in Figure 2a. With the increase of the iteration, the number of clusters reduce one each time, and the optimal segmentation can be obtained under the condition of each number of clusters shown in Figure 2b–h. For the homogeneous region with smaller scale parameters like region I, it is easy to be segmented, and the heavily interwoven regions like regions II and III must be divided by iterations with the help of MRF. In the iterations, the global energy value *L* and the number of clusters *m* with iteration *τ* are shown in Figure 3. Figure 3a,b are the full view and local amplification image. It can be seen that the minimum global energy function value is 8.15465 × 10^4^ corresponding to the iteration 6, and the corresponding number of clusters is 4. Thus, the final optimal segmentation result is the one shown in Figure 2f.

**Figure 4:**
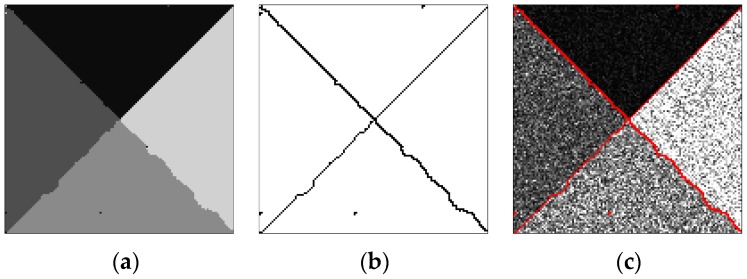
Segmentation results of the proposed algorithm. (**a**) Final optimal result; (**b**) Outline; (**c**) Superposition.

The final optimal segmentation results by the proposed algorithm are displayed in Figure 4. Figure 4a is the final optimal segmentation, Figure 4b is the outline of the homogeneous regions, and Figure 4c shows the result of outlines overlaid on the original image.

**Figure 5:**
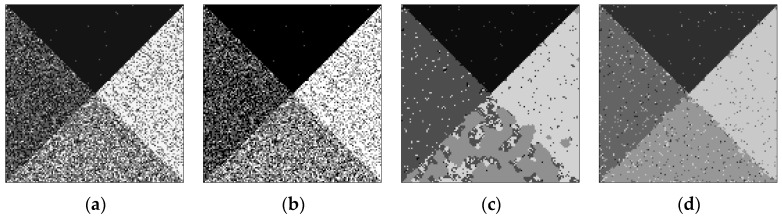
Segmentation results of comparing algorithms with (**a**) ISODATA; (**b**) AGNES; (**c**) HMRF-FCM; (**d**) Gamma-MRF.

One can see from Figure 4 that the proposed algorithm can segment each homogeneous region well. There are almost no misclassified pixels, and the boundary is smooth and accurate. For qualitative analysis of the proposed algorithm, the segmentation results of the comparing algorithms are displayed in Figure 5, where Figure 5a–d are the segmentation results of ISODATA, AGNES, HMRF-FCM and Gamma-MRF, respectively. It shows that the ISODATA and AGNES algorithms can find the right number of clusters but ISODATA need more prior condition to limit the range of results and the result is pretty bad. Although AGNES avoids the help of prior knowledge, its segmentation effect is equivalent to ISODATA, there are many misclassified pixels in Figure 5a,b. The HMRF-FCM algorithm can reduce the influence of noise by introducing MRF to model the relation between neighbor pixels. It still cannot distinguish the homogeneous region well. For the Gamma-MRF algorithm, the Gamma distribution combining MRF theory can accurately describe the distribution of pixel intensities, but there are also many pixel errors. From the visual viewpoint, the proposed algorithm is much better than the compared ones.

**Figure 6:**
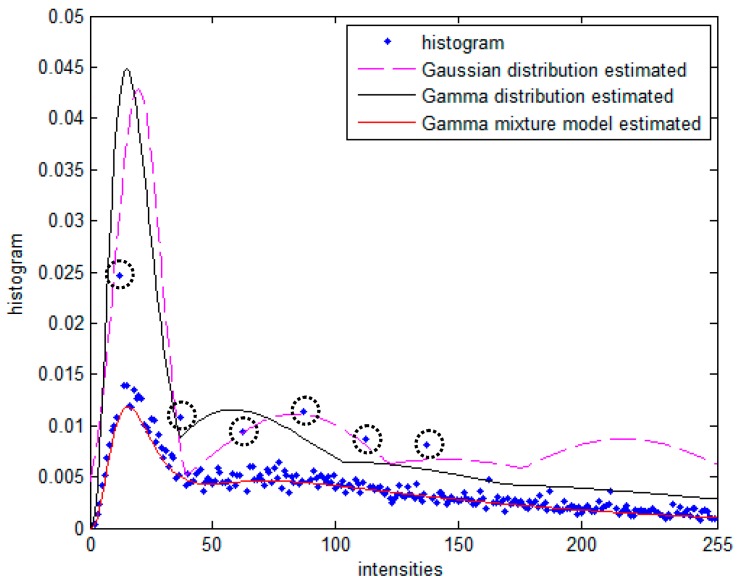
Histogram for simulated image and estimated curve with Gaussian distribution, Gamma distribution and Gamma mixture model.

In order to demonstrate the superiority of GaMM, the histogram for simulated image and the estimated curve with Gaussian distribution, Gamma distribution and GaMM, respectively, are shown in Figure 6. From Figure 6, one can see that the GaMM with different weights can fit the histogram of the image more accurately.

To evaluate the proposed and comparing algorithms in quantitative, the main parameters, running time, accuracy and Kappa coefficient are shown in Table 2. For the proposed algorithm, the running time is 1139.19 s. The user’s accuracy and producer’s accuracy of each homogeneous region are more than 98%, and the overall accuracy and kappa coefficient are 99.34% and 0.99, respectively. For the comparing algorithms, the producer’s accuracy of region III is only 36.16% and 40.28 % in ISODATA and AGNES, and the overall accuracy and kappa coefficient are about 60% and 0.5, respectively. HMRF-FCM and Gamma-MRF are better than the two algorithms discussed above, but the accuracy is still lower than that for the proposed algorithm. Generally, the running time of statistical model is usually much higher than clustering methods, but compared with Gamma-MRF, the proposed algorithm greatly reduces the time. Thus, Table 2 precisely describes the effectiveness and robustness of the proposed algorithm.

Furthermore, the mean and variance listed in Table 3 are also used to evaluate the quality of the proposed algorithm. One can see that the deviation rates between the estimated and actual values for all homogeneous regions are very small. Table 3 illustrates the accuracy of the algorithm.

In order to analyze the robustness of the method and the segmentation accuracy affected by different span parameter *d* and neighborhood coefficient *η*, the relationship between the number of clusters, overall accuracy, running time and span parameter *d*, neighborhood coefficient *η* are given in Table 4. When the neighborhood coefficient is small, the number of clusters obtained are less than the real one. On the contrary, it is larger than the real one. In this part, we only consider the overall accuracy under the correct number of clusters. We can see that the accuracy of *d* = 30 is almost higher than others under the same neighborhood coefficient. In Table 4, when *d* = 30, *η* = 0.8, the overall accuracy is the highest.

**Figure 7:**
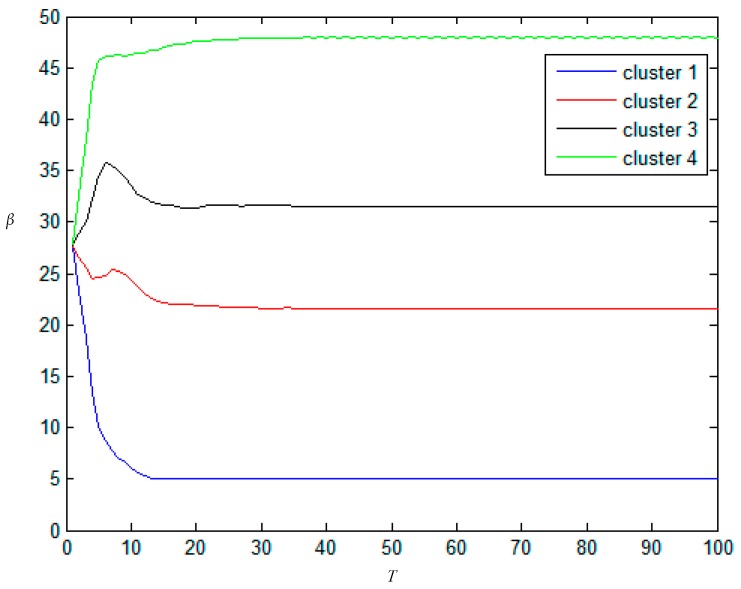
The changing curve of scale parameters in each clusters in inner loop iterations.

In order to prove that *T* = 20 is the optimal result, the change of scale parameter *β* with iteration is shown in Figure 7. From Figure 7 one can see that scale parameter *β* for each cluster converges to its stable value after 20 iterations. Consequently, 20 is regard as *T*’s optimal choice, because it not only ensures the accuracy of segmentation but also saves time. Furthermore, the sensitivity of the value of *T* is also analyzed through the segmentation accuracy with different *T* under *d* = 30, *η* = 0.8, the results are listed in Table 5. If the value of *T* is too small, the segmentation result is inaccurate, if the value of *T* is too large, the running time will be increased.

### 3.2. Real SAR Images Segmentation

**Figure 8:**
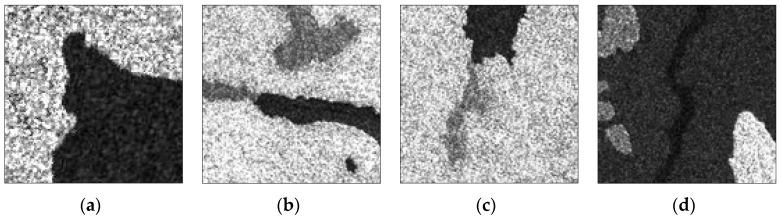
Original real SAR images. (**a**–**d**) are sea ice images with 2, 3, 3, 4 clusters.

Figure 8 shows the real SAR images with size of 128 × 128 pixel and the real number of clusters are 2, 3, 3 and 4, respectively. Figure 8a is a RADARSAT-I intensity image with HH polarization and the spatial resolution of 30 m, Figure 8b,c are RADARSAT-II images with VV polarization and the spatial resolution of 25 m, Figure 8d is a RADARSAT-I image with HH polarization and the spatial resolution of 25 m. All of them are sea ice images in different periods, from light to dark, representing sea ice with density from high to low. The image in Figure 8a with two clusters is easy to segment for all algorithms. For the images in Figure 8b,c with three clusters, it is difficult to segment the different types of sea ices. For the image in Figure 8d with four clusters, the difficulty is that the water is easy to ignore because its area is too small compared with the background of low density sea ice areas.

**Figure 9:**
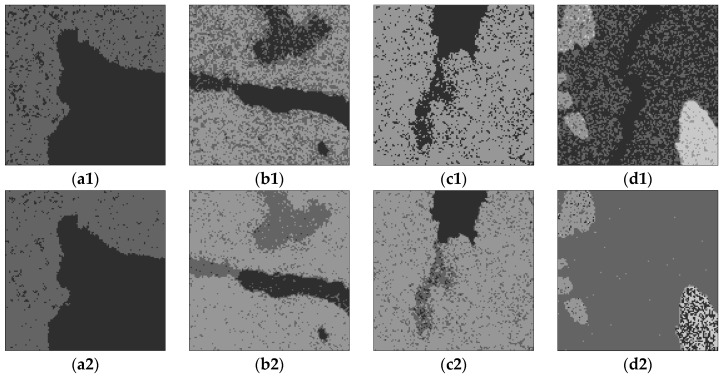

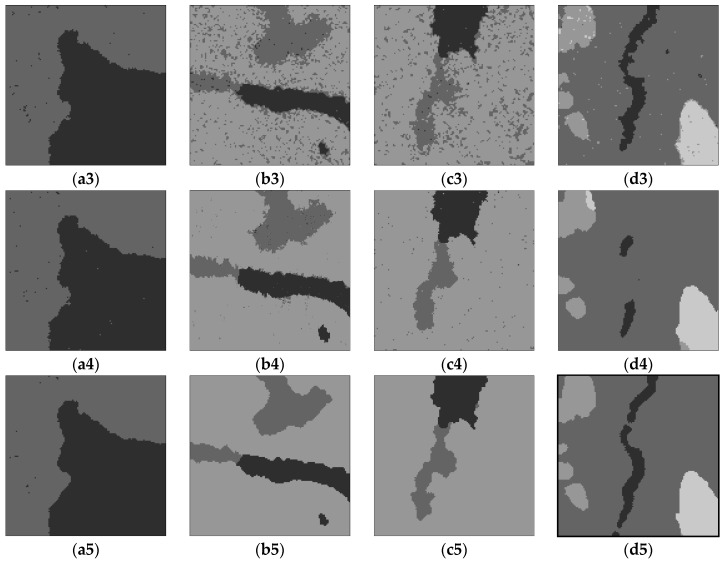
Segmentation results of real SAR image with the proposed and comparing algorithms. (**a1**–**d1**) ISODATA; (**a2**–**d2**) AGNES; (**a3**–**d3**) HMRF-FCM; (**a4**–**d4**) Gamma-MRF; (**a5**–**d5**) The proposed algorithm.

Figure 9 is the segmentation results with the proposed algorithm and the comparing algorithms of ISODATA, AGNES, HMRF-FCM and Gamma-MRF. Figure 9(a1–d1) are the results of the ISODATA algorithm, Figure 9(a2–d2) are the results of the AGNES algorithm, Figure 9(a3–d3) are the results of the HMRF-FCM algorithm, Figure 9(a4–d4) are the results of the Gamma-MRF algorithm and Figure 9(a5–d5) are the results of the proposed algorithm.

It can be seen that the ISODATA algorithm cannot always determine the right number of clusters, and as shown as Figure 9(c1), the real number of the original image is 3 while the estimated number is 2, which is a fatal error for image segmentation.

Although the AGNES algorithm can find the right number of clusters, it cannot overcome the noise effect well and ignores the existence of water class, see Figure 9(d2).

Although the HMRF-FCM algorithm combines a fuzzy cluster based on Gaussian distribution and MRF, it also cannot obtain a satisfactory segmentation result.

The Gamma-MRF algorithm adopts the Gamma distribution to describe the data, and its results are not good enough either. Besides, both HMRF-FCM and Gamma-MRF lack a mechanism for changing the number of clusters, which makes the algorithm not flexible enough in applications.

As shown in Figure 9(a5–d5), the segmentation results from the proposed algorithm are all the optimal segmentation results under a real number of clusters. One can see that the sea ice images during different periods are evidently distinguished all the time by the proposed algorithm, and there are almost no mis-segmentations in the homogeneous regions.

**Figure 10:**
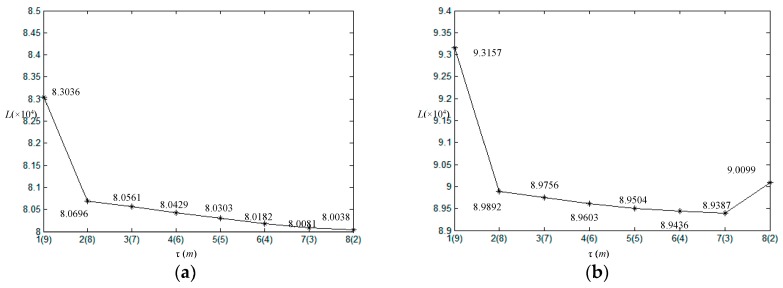

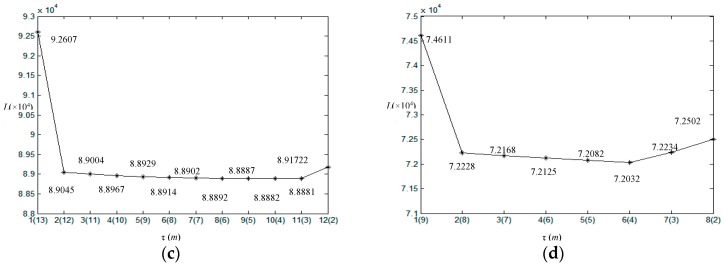
Global energy value *L* and the number of clusters *m* at each iteration. (**a**) Original SAR image 1 with *d* = 30, *η* = 0.5; (**b**) Original SAR image 2 with *d* = 30, *η* = 0.4; (**c**) Original SAR image 3 with *d* = 20, *η* = 0.7; (**d**) Original SAR image 4 with *d* = 30, *η* = 0.5.

In addition, the relation of the given parameter, global energy value and the number of clusters are shown in Figure 10. The minimum global energy value is 8.00387 × 10^4^, 8.93877 × 10^4^, 8.88819 × 10^4^ and 7.20323 × 10^4^ corresponding to the iterations 8, 7, 11, 6, and the corresponding number of clusters are 2, 3, 3, 4, respectively. It illustrates that Figure 9(a5–d5) with the proposed algorithm under the real number of clusters are the perfect results.

The values of estimated parameters and running time for real images segmentation results are shown in Table 6. Combining with the results in Figure 9 and the running time in Table 6, it can be seen that the running time of the proposed algorithm has a high cost performance.

**Figure 11:**
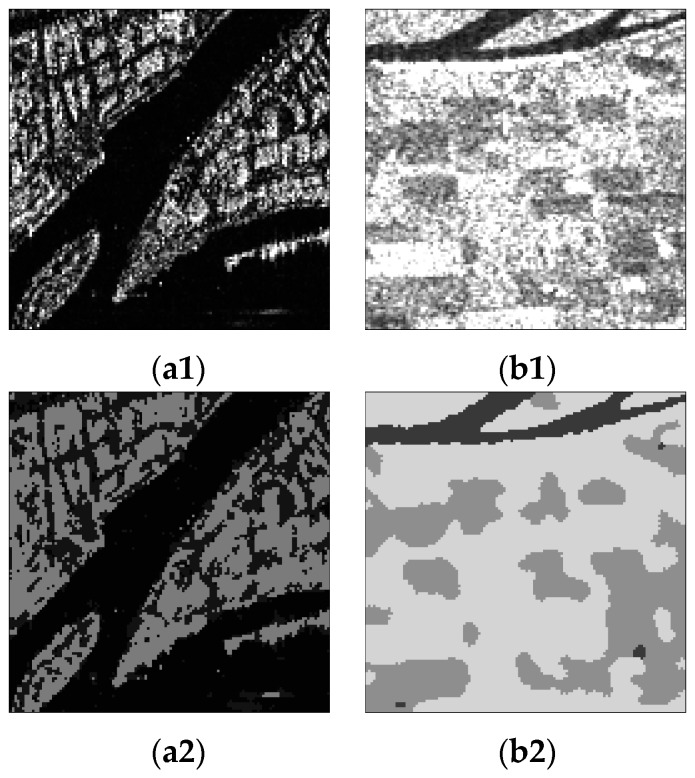
Segmentation results of urban and agricultural SAR images with the proposed algorithm, where (**a1**,**b1**) are the original SAR images, (**a2**,**b2**) are the segmentation results from the proposed algorithm.

In addition, segmentation experiments for SAR images covering urban and agricultural areas were also carried out. Figure 11(a1,b1) show the HH RADARSAT-I/II intensity images for an urban area with two looks and an agricultural area with three looks, respectively. The scene shown in Figure 11(a1) contains three objects, including water, streets and buildings, and the scene in Figure 11(b1) contains water and two kinds of farmland. Figure 11(a2,b2) are the corresponding segmentation results from the proposed algorithm with *d* = 20, *η* = 0.3, *α* = 2 and *d* = 20, *η* = 0.35, *α* = 3, respectively. One can see that the proposed algorithm can separate the street from water on the basis of segmenting buildings and effectively distinguish different kinds of farmland. The water and streets shown in Figure 11(a2) are not clearly displayed, just because their grayscales are similar. Figure 11 illustrates that the proposed algorithm can segment not only simple SAR images well, but also complex images with different noise levels.

## 4. Conclusions

In this paper, a novel hierarchical clustering segmentation algorithm for multilook SAR image segmentation is proposed. The GaMM can describe the statistical characteristics of the image more accurately than unimodal character model. The hierarchical clustering with global energy merging criterion can determine the real number of clusters and obtain the accurate segmentation results simultaneously. The qualitative and quantitative analyses of the simulated and real images with the proposed and comparing algorithms fully demonstrate the superiority of the proposed algorithm in multilook SAR image segmentation. Some improvements of this research are still needed. On the one hand, the coarse segmentation method could be replaced with some more efficient approaches, such as, Fuzzy C-means (FCM). On the other hand, the image segmentation model can be improved by considering texture features.

## Figures and Tables

**Table 1:** Gamma distribution parameters for each homogeneous region.

Parameters	Homogeneous Region			
	I	II	III	IV
*α*	4	4	4	4
*β*	5	20	35	65

**Table 2:** Main parameters, running time, accuracy and Kappa coefficient of simulated image.

Algorithm	Main Parameter	Value	Time (s)	Accuracy	Homogeneous Region			
					I	II	III	IV
The proposed	span parameter *d*	30	1139.19	user’s	99.76	99.42	99.50	98.67
				producer’s	100	99.57	98.14	99.70
	neighborhood coefficient *η*	0.8		overall	99.34			
				Kappa	0.99			
ISODATA	expected number of clusters	4	5.08	user’s	69.29	52.83	50.67	77.41
	minimum number of pixel	2500		producer’s	99.61	54.01	36.16	63.74
	upper bounds of standar *d* deviation	10		overall	63.34			
	lower limit of distance	10		Kappa	0.51			
AGENES	span parameter *d*	30	30.77	user’s	67.86	53.19	49.62	79.08
				producer’s	99.93	50.46	40.28	60.64
				overall	62.74			
				Kappa	0.50			
HMRF FCM	fuzzy coefficient	0.5	32.4449	user’s	98.31	80.32	95.16	82.16
				producer’s	99.19	96.03	60.99	94.79
	neighborhood coefficient	1		overall	87.76			
				Kappa	0.84			
Gamma MRF	neighborhood coefficient	0.2	5902.28	user’s	96.98	93.02	92.01	92.06
				producer’s	98.75	90.96	88.45	96.06
	shape parameter	4		overall	93.54			
				Kappa	0.91			

**Table 3:** Comparison of the estimated and actual values of mean and variance.

Parameters		Homogeneous Region			
		I	II	III	IV
mean	actual/estimated	19.90/19.87	79.24/79.39	137.07/137.02	208.95/208.45
	deviation rate	−0.0015	0.0019	−0.0004	−0.0024
variance	actual/estimated	9.96^2^/9.89^2^	39.09^2^/39.07^2^	61.40^2^/61.16^2^	57.53^2^/57.92^2^
	deviation rate	−0.0140	−0.0010	−0.0078	0.0136

**Table 4:** The number of clusters and overall accuracy of segmentation with different *d* and *η*.

*d*	*η*										Approximate Time (s)
	Number of Clusters/Overall Accuracy										
	0.1	0.2	0.3	0.4	0.5	0.6	0.7	0.8	0.9	1.0	
10	3	3	4/94.73	4/97.06	4/97.33	4/97.45	4/97.39	4/97.99	4/97.56	6	21,600
20	3	3	4/93.61	4/96.05	4/96.60	4/97.66	4/98.18	5	5	6	3000
30	3	3	4/94.62	4/97.59	4/98.29	4/99.04	4/98.84	4/99.34	6	6	1000
40	3	3	4/93.38	4/93.71	4/97.78	4/98.31	4/98.72	4/98.60	6	6	800
50	3	3	4/93.21	4/96.63	4/97.52	4/97.92	4/98.00	5	5	5	500

**Table 5:** The running time, number of clusters and overall accuracy of segmentation with different *T*.

Parameters	*T*				
	1	5	20	30	50
number of clusters/accuracy	6	4/99.15	4/99.34	4/99.34	4/99.34
time (s)	305.687	435.110	1139.19	1150.107	1266.915

**Table 6:** Main parameters and running time of real images.

Algorithm	Main Parameter	Image 1		Image 2		Image 3		Image 4	
		Value	Time (s)	Value	Time (s)	Value	Time (s)	Value	Time (s)
The proposed	span parameter *d*	30	1387.64	30	1450.60	20	2099.92	30	1192.03
	neighborhood coefficient *η*	0.5		0.4		0.7		0.5	
	inner loop *T*	20		20		20		20	
	shape parameter	4		4		4		4	
ISODATA	expected number of clusters	2	2.96	3	4.01	3	3.37	4	5.42
	minimum number of pixel	5000		3000		3000		1000	
	upper bounds of standard deviation	10		20		10		10	
	lower limit of distance	10		20		10		10	
AGENES	span parameter *d*	30	3.48	30	5.12	30	9.17	30	5.02
HMRF FCM	fuzzy coefficient	0.5	2.84	0.1	5.8286	0.5	6.17	0.5	8.60
	neighborhood coefficient	0.7		4		0.75		0.95	
Gamma MRF	neighborhood coefficient	0.2	4226.35	0.2	10,593.52	0.4	10,367.17	0.34	9378.35
	shape parameter	4		4		4		4	
